# Differences in Epstein-Barr Virus Characteristics and Viral-Related Microenvironment Could Be Responsible for Lymphomagenesis in Children

**DOI:** 10.3390/pathogens9010068

**Published:** 2020-01-19

**Authors:** Aldana Vistarop, Oscar Jimenez, Melina Cohen, Elena De Matteo, Maria Victoria Preciado, Paola Chabay

**Affiliations:** 1 Multidisciplinary Institute for Investigation in Pediatric Pathologies (IMIPP), CONICET-GCBA. Molecular Biology Laboratory, Pathology Division, Ricardo Gutiérrez Children’s Hospital, Buenos Aires C1425EFD, Argentina; avistarop@hotmail.com (A.V.); osky_30@hotmail.com (O.J.); melucohen_82@yahoo.com.ar (M.C.); preciado@conicet.gov.ar (M.V.P.); 2 Multidisciplinary Institute for Investigation in Pediatric Pathologies (IMIPP), CONICET-GCBA. Pathology Division, Ricardo Gutiérrez Children’s Hospital, Buenos Aires C1425EFD, Argentina; eledm9@hotmail.com

**Keywords:** EBV, Hodgkin lymphoma, germinal center, microenvironment, children

## Abstract

In Argentina, Epstein-Barr virus (EBV) presence is associated with Hodgkin lymphoma (HL) in patients younger than 10 years, suggesting a relationship between low age of EBV infection and HL. Given that HL is derived from germinal centers (GC), our aim was to compare EBV protein expression and microenvironment markers between pediatric HL patients and EBV+GC in children. Methods: EBV presence and immune cell markers were assessed by in situ hybridization and immunohistochemistry (IHC). Results: Viral latency II pattern was proved in all HL patients and in 81.8% of EBV+ tonsillar GCs. LMP1 and LMP2 co-expression were proved in 45.7% HL cases, but only in 7.7% EBV+ GC in pediatric tonsils. An increase in CD4+, IL10, and CD68+ cells was observed in EBV+ GC. In pediatric HL patients, only the mean of IL10+ cells was statistically higher in EBV+ HL. Conclusions: Our findings point us out to suggest that LMP1 expression may be sufficient to drive neoplastic transformation, that an immune regulatory milieu counteracts cytotoxic environment in EBV-associated Hodgkin lymphoma, and that CD4+ and CD68+ cells may be recruited to act in a local collaborative way to restrict, at least in part, viral-mediated lymphomagenesis in tonsillar GC.

## 1. Introduction

The infectious etiology of Hodgkin lymphoma (HL) has been described a long time ago, based on epidemiological evidence that demonstrates a bimodal age-specific incidence pattern that can be observed throughout the western world [1]. A hallmark of HL is the presence of Hodgkin Reed Sternberg (HRS) tumor cells surrounded by various inflammatory immune cells. Therefore, the attraction of inflammatory cells plays an important contribution to the development of HL [2]. The reactive cells include lymphocytes, macrophages, eosinophils, mast cells, plasma cells, stromal cells, fibroblasts, and other cells. The clinical and pathologic features of HL reflect an abnormal immune response, which alters the composition and function of the cells in the surrounding microenvironment and shapes the specific histopathologic appearance of the lymph node. The most abundant cells found in HL are infiltrating CD4+ T cells, which mainly display T helper 2 (Th2) and T regulatory (Treg) phenotypes [3].

HL is one of the Epstein-Barr virus (EBV)-related malignancies. Nearly all HRS cells in EBV+ HL cases carry the latent form of viral infection, indicating EBV infection as an early, primary event in the development of the disease [4]. EBV rates in HL from North America and Europe, as well as from other developed populations, have been shown to vary between 20% and 50%, whereas much higher rates are observed in underdeveloped countries, suggesting a contribution of socio-economic factors to the pathogenesis of EBV-associated cases of HL [5]. In Argentina, an EBV association of 31% was described for adult HL, whereas EBV presence rose up to 54% in pediatric patients. However, in the pediatric HL group, EBV association was remarkably observed in children younger than 10 years old, in males and in mixed cellularity subtype, typical of an underdeveloped or developing population [6]. The latent viral proteins expressed in HRS cells are the EBV nuclear antigen 1 (EBNA1), which is essential for replication of the viral genome in proliferating cells, together with the two latent membrane proteins, LMP1 and LMP2A. LMP1 mimics an active CD40 receptor while LMP2a mimics a B cell receptor (BCR), thus providing two essential signals for the survival of germinal centers (GC) B cells, the normal counterpart of HRS cells [4].

Germinal centers (GCs) are the histological structures dedicated to the generation and the selection of B cells that produce high-affinity antibodies. However, the same genetic mechanisms that enable the development of high-affinity immunoglobulin receptors are involved in the malignant transformation of B cells. Therefore, GC B cells represent the main transformed cells of most mature B cell lymphomas [7]. There are two models to explain how the virus enters memory B cells, the site of viral persistence. In the germinal center model, EBV infection of naive B cells leads to the latency III (LIII) or growth program, in which the proliferation and expansion of the infected B cell pool are driven by expression of all EBV latent genes. Afterward, infected B cells enter the GC and express the latency II (LII) or default program, characterized by the expression of EBNA1, LMP1, and LMP2A. Finally, they leave the GC as infected memory B cells with all EBV protein expression silenced (latency 0 or L0). In the direct infection model, EBV accesses memory B cells through direct infection of these cells, which may involve an LIII intermediary [8]. LMP1 and LMP2A have been proposed to alter B cell functions including the ability of latently-infected B cells to access and transit the GC, since LMP2A plays a role in the modulation of LMP1 signaling. In fact, LMP2A expression in LMP1+/2A+ animals rescued the impairment in GC generation promoted by LMP1 single expression [9]. The frequency of EBV-infected GC B cells in normal, persistently-infected, individuals is very low, in fact, most viral antigens are located at the interfollicular (IF) region [10], whereas viral loads in memory B cells are higher than those found in GC B cells [11]. The GC model provides an explanation for the origin of EBV-associated lymphomas. In fact, the EBV LII or default program observed in infected GC B cells is also expressed in HL, probably the stage of B cell differentiation from which the EBV-positive HL arises [8].

The HL microenvironment contributes to disease pathogenesis, not only by stimulating tumor cell growth and survival, but also potentially by redirecting the functions of the EBV latent proteins expressed by HRS cells [12]. Tumor microenvironment, particularly T cell, B cells, and macrophages, has emerged as a crucial player in the pathogenesis of human lymphomas. Abnormal microenvironmental factors are involved in tumor development, cell growth, and lymphoma progression [13]. EBV influence on microenvironment composition has been characterized in HL, in which the coexistence of functional Th1 cell infiltrate with T regulatory (Tregs) cells, in the context of an increased frequency of activated CD8+ T cells and natural killer (NK) cells were described [14]. Furthermore, a pronounced infiltration of macrophages associated with EBV presence was also illustrated [14]. The microenvironment composition around EBV tonsillar infected cells at the GC of the asymptomatic host to become a malignant cell is still not disclosed. Moreover, we hypothesize that a failure in immune response around EBV infected cells could be involved in EBV mediated transformation as a key factor in HL pathogenesis. Therefore, our aim is to compare microenvironment markers around EBV+ HRS cells and EBV+ GC cells in children with HL and asymptomatic infection, respectively. This characterization may shed light on EBV-associated pediatric HL pathogenesis.

## 2. Results

**Figure 1:**
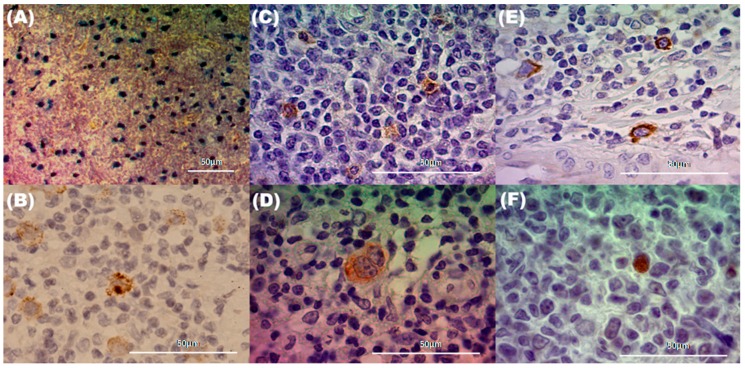
LMP1 and EBERs expression in tonsil and Hodgkin lymphoma (HL) Epstein-Barr virus (EBV)+ pediatrics patients. (**A**) EBERs-specific in situ hybridization with RNA probes revealed specific black–dark blue nuclear staining in Hodgkin Reed Sternberg (HRS) of pediatric HL; (**B**) LMP1 membranous and cytoplasmic positive staining in HRS at pediatric HL and (**C**) germinal centers (GC) positive lymphocytes in tonsil; LMP2A membranous and cytoplasmic positive staining in (**D**) pediatric HL and (**E**) at the GC in tonsil; (**F**) EBNA2 nuclear positive staining in tonsil lymphocyte.

The aim of this study was to compare EBV viral protein expression and microenvironment composition in pediatric HL samples, a tumor derived from GC lymphocytes, with normal tonsil GCs from pediatric patients preliminary described [15] as a counterpart. EBV presence and viral protein expression were analyzed in 60 pediatric HL patients and 55 tonsils from children undergoing tonsillectomy by EBERs in situ hybridization (ISH) and immunohistochemistry (IHC) for viral latency proteins (LMP1, LMP2A, and EBNA2). Both EBERs positive ISH along with LMP1 expression exclusively at the tumor HRS cells were found in 43/60 (71.7%) of HL cases (Figure 1A,B). Given that we have previously observed in the tonsils samples that all cases expressing EBERs also showed LMP1 positivity [10], the viral presence was assessed at the tonsils by LMP1 expression, which was observed in every tonsil (Figure 1C). Interestingly the LMP1 labeling pattern could be classified into two groups: the GC region and outside the GC, which included the interfollicular (IF) and subepithelial regions.

It is largely known that age has a great influence on EBV infection. Therefore, age characteristics were analyzed in both populations. The mean age difference was statistically significant between EBV+ HL cases (8 years) and the EBV− ones (11 years) (*p* = 0.0024, Mann–Whitney test). LMP1 expression was found in the GC and outside the GC, but there was no difference in the mean age of patients disclosing each pattern (*p* = 0.3139, Mann–Whitney test).

Latency profile was defined by LMP1 and EBNA2 expression, as follows: LI, cases with EBERs expression; LII, LMP1+ cases without EBNA2 expression; and LIII, LMP1+ cases along with EBNA2 expression. As expected, the complete series (100%) of pediatric HL patients displayed LII pattern, given that all cases were LMP1+ and EBNA2−. In contrast, in pediatric tonsil, 45/55 (81.8%) cases displayed LMP1 positive cells specifically located at the GC, and 10/55 (18.2%) were located exclusively outside the GC. Interestingly, in 8/45 LMP1+ GC, EBNA2 expression was also observed at the same location in those cases. Therefore, the LII pattern was described in 37/45 cases at GC (82%), while the remaining 8/45 (18%) cases displayed LIII pattern. Furthermore, only nine cases exhibited EBNA2 expression, in eight cases located in both GC and outside the GC, whereas in one case EBNA2 expression was placed outside the GC, at the IF region, indicating that EBNA2 expression and LIII pattern were located by and large at the GC region (Figure 1D–F).

It was previously suggested that LMP1 and LMP2, when co-expressed in vivo at the GC, can modulate each other’s signaling [13]. In order to determine if the expression of LMP2A is involved in the transit of LMP1+ lymphocytes throughout the GC, joint expression of LMP1 and LMP2A was analyzed according to its location within CG or outside of it. Therefore, LMP2A expression was performed in a subset of 39 tonsils and 35 HL available cases, selected based on the availability of sufficient formalin-fixed, paraffin-embedded tissue for analysis. Concerning HL, which is derived from GC B lymphocytes, 16 (45.7%) cases expressed both LMP1 and LMP2A latency antigens, whereas in 19 cases (54.3%) only LMP1 expression was proven. Among 39 tonsils, 23 cases (59.0%) were LMP1+/LMP2A+ and 16 (41.0%) displayed LMP1+/LMP1− pattern. Only three (7.7%) cases showed LMP1+ along with LMP2A+ cells at the GC, while in the remaining 20 (51.2%) cases LMP1+/LMP2A+ cases, in 18 (46.2%) LMP2A+ cells were outside the GC and in two (5.1%) cases both LMP1+ and LMP2A+ cells were located outside the GC. On the other hand, 16 cases were LMP1+/LMP2A− cases, most of which (13, 33.3% of 39 tonsils) were located at the GC. Therefore, a statistical association was observed regarding the expression of both proteins outside the GC, whereasLMP1+ cells in absence of LMP2A prevailed in the CG (*p* < 0.0001, Fisher’s exact test). In order to deeply characterize EBV expression at the GC, LMP1 positive cells were counted at the GC and then compared with LMP1 positive cells outside the GC. The number of LMP1+ cells was statistically higher outside the GC than at the GC (*p* < 0.0001, Mann–Whitney test). 

**Figure 2:**
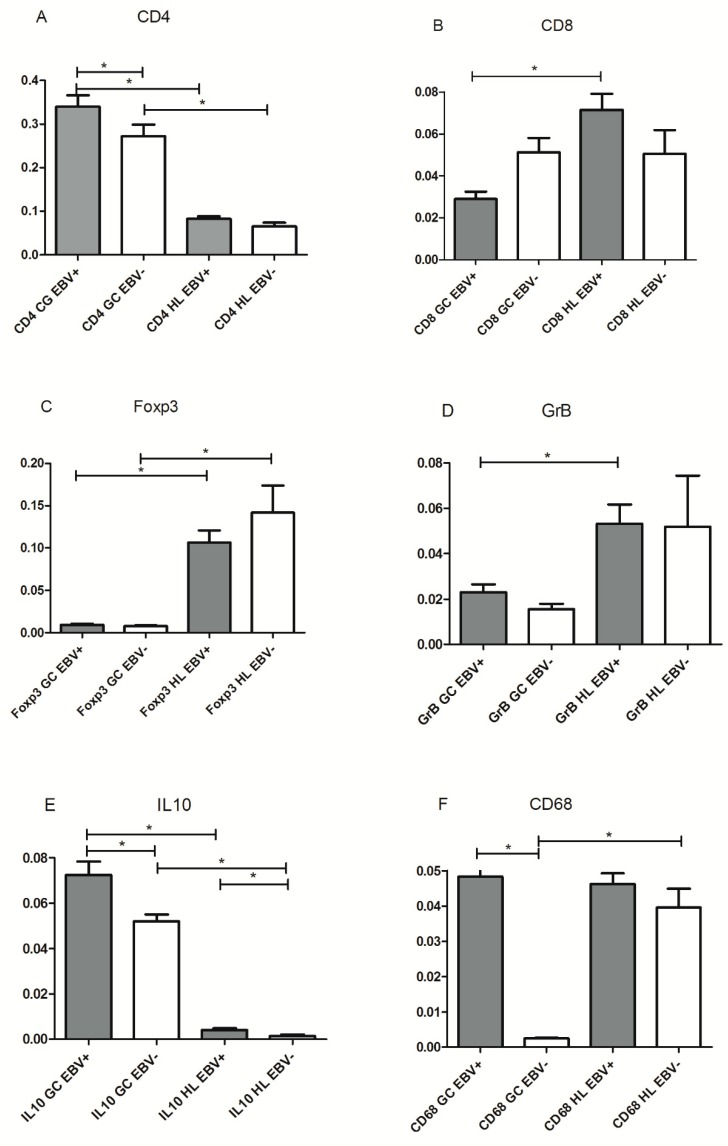
Comparison of immune cell markers (**A**) CD4; (**B**) CD8; (**C**) Foxp3; (**D**) GrB; (**E**) IL10; (**F**) CD68; analyzed in relation to EBV status, in tonsils and HL. Dark grey bars indicate EBV+ region and white bars indicate EBV+ region. The *p*-value is from the Wilcoxon test or Mann–Whitney test when appropriate (* *p* < 0.05).

In EBV-associated HL, an increase in activated CD8+ T cells cell numbers and macrophages, in the context of a regulatory Treg+ microenvironment was described [14]. Therefore, CD8+ and GrB+ T cells (as markers of activated cytotoxic cells), CD4+, Foxp3+, and IL10+ cells (as markers of regulatory milieu), and CD68+ cells (as markers of macrophage infiltration) were assessed, in order to compare microenvironment around EBV+ HRS cells and EBV+ GC cells. Initially, with the aim of characterizing LMP1 expression and the immune composition around EBV tonsillar infected cells, EBV+ and EBV− GC were defined by means of LMP1 expression, and microenvironment composition around infected and noninfected zones within EBV+ cases was compared. Once those zones were defined, serial slides were stained for CD4, CD8, Foxp3, GrB, IL10, and CD68 for microenvironment characterization. CD8+, GrB+, and Foxp3+ cell counts at the GC did not show statistical differences between LMP1+ and LMP1− zones (*p* > 0.05, Wilcoxon test). However, a statistical increase in CD4+, IL10+, and CD68+ cells was observed in LMP1+ GC compared with the LMP1− ones (*p* = 0.0002, *p* = 0.0013, and *p* < 0.0001, respectively, Wilcoxon test). Furthermore, LMP1+ cell numbers at the CG displayed a statistically negative correlation with CD4+ (r = −0.388, *p* = 0.004, Spearman correlation test), whereas this correlation was not proved for CD68 and IL10 (r = −0.022, r = −0.015, respectively, *p* > 0.05, Spearman correlation test). In contrast, in pediatric HL patients neither CD8+, GrB+, Foxp3+ mean cell counts nor CD4+ and CD68+mean cell counts showed statistical differences when EBV+ cases were compared with the EBV− counterpart (*p* > 0.05, Mann–Whitney test). Remarkably, only the mean of IL10+ cells was statistically higher in EBV+ pediatric HL cases compared to EBV− ones (*p* = 0.0272, Mann–Whitney test) (Figure 2). 

In order to evaluate if the malignant context contributed to EBV influence on immune cell composition around EBV-infected cells, each immune cell marker analyzed was compared between GC and HL environments in both EBV+ and EBV− cases, in the complete series (60 pediatric HL patients and 55 tonsils from children undergoing tonsillectomy). CD4+, Foxp3+, CD68+, and IL10+ mean cell counts displayed statistical differences when GC and HL microenvironments were compared in both EBV+ and EBV− cases (*p* < 0.05, Mann–Whitney test). However, CD8+ and GrB+ cell counts were statistically higher in EBV-associated HL compared to EBV+ GC, whereas this difference was not proved in EBV− cases (*p* < 0.05, Mann–Whitney test) (Figure 2).

Given the fact that it was previously demonstrated that CD4+ T cells are involved in the control of the EBV-induced B cell proliferation by EBNA2 downregulation by soluble factors [16], CD4+ cell count was compared between GC with EBNA2+ cells and those without them, and with LMP2A cells and without them. Neither EBNA2 nor LMP2A expression had an effect on CD4+ T cell counts at the tonsillar GC (*p* > 0.05, Mann–Whitney test). In line with this, LMP2A expression displayed no effect on CD4+ T cell number in pediatric HL cases (*p* > 0.05, Mann–Whitney test), whereas EBNA2 was not expressed in HL patients. 

## 3. Discussion

GC is the histological structure dedicated to the generation and the selection of B cells that produce high-affinity antibodies. However, the same genetic mechanisms that enable the development of high-affinity immunoglobulin receptors of different isotype classes are involved in the malignant transformation of B cells. Therefore, GC B cells represent the normal counterpart of many mature B cell lymphomas [7]. Once naive B cells are infected by EBV, they begin to proliferate in response to the nine viral latent proteins action and then migrate toward the GC, where infected B cells are truly undergoing a GC reaction [9]. It has been demonstrated that there is a low frequency of EBV-infected GC B cells in normal persistently-infected individuals [17], while LMP1+ cells number at the outside of the GC was higher than the cell counts at the GC in our series as well. In fact, previous studies that used EBERs in situ hybridization to study EBV presence in the lymph nodes of infected individuals have shown that EBER+ cells are mainly located in the IF region [18]. Furthermore, it was suggested that an increase of EBV-positive B cells in GC is observed in patients with a history of immunosuppression [12].

In spite of low LMP1+ cell numbers demonstrated at the GC in tonsil samples, the EBV+ cells express the LII or default program, which involves only three of the nine latent proteins, EBNA1, LMP1, and LMP2A, which is the same pattern observed in HL [19]. In line with this observation, the LII pattern depicted in all HL cases was also proved in this series. However, the scenario was quite different in tonsillar GC from children infected with EBV, given the fact that the LII pattern at the GC was described in 82% of cases, while the remaining 18% of cases displayed the LIII pattern. Even though the EBV infection of naïve B cells followed by the transit throughout the GC has been demonstrated [9], EBV antigen expression at this histological site remains controversial. Mohamed et al. did not observe LMP1 or LMP2 expression in EBER+ GC, suggesting that the expansion of EBV+ B cells was driven by antigen rather than by LMP1/2 signaling [12]. In contrast, Kurth et al. proved that EBV-infected cells in GCs exhibited an unusual EBV expression pattern in that they were positive for EBNA2 but negative for LMP1 [20]. The expression of EBNA proteins, in the absence of LMPs, was referred to as LIIb, and demonstrated in early infected cells [21]. LMP1 along with EBNA2+ cells were described in a few non-infectious mononucleosis (IM) tonsils from adult EBV carriers, mostly restricted to the IF region [22]. Even though EBNA2 interferes with the GC phenotype by downregulation of the GC hallmark genes, particularly BCL-6 [23], the presence of EBNA2 at the GC in our series argues against this previous observation. In addition, the absence of EBNA2 in our HL series may suggest, on one hand, that EBNA2 is not involved in the pediatric lymphomagenesis process, or, quite the opposite, that it may participate in the first steps of malignant transformation at the GC, to be turned off afterward.

The collaborative actions of LMP1 and LMP2 in the LII program are to provide the two signals, T cell help, and BCR, respectively, necessary to rescue the GC cell into memory, by modulating each other’s signaling. In fact, LMP2A expression in latently infected cells would ensure the GC formation, LMP1 and LMP2A together drive class switch recombination and somatic hypermutation and provide the survival signals, and then LMP1 alone ensures exit from the GC by switching off BCL-6 and switching on BCL-2. HRS cells arise from EBV-infected cells expressing the LMP1+/LMP2A+ LII program [9]. Specifically, at the GC in tonsils from pediatric carriers, only a few cases expressed both LMP1 and LMP2A antigens together, while in many cases LMP1+/LMP2A+ cells were observed outside the GC. In addition, only 45.7% of pediatric HL cases co-expressed LMP1 and LMP2A, whereas the rest exhibited LMP1 presence exclusively. This fact may suggest that the presence of LMP1 and LMP2A together is not an exclusive requisite for lymphoma development in those patients, and that LMP1 expressed alone could be the driving force of malignant transformation HL cells derived from GC. This finding is reinforced by the presence of LMP1+ and LMP2A+ cells mostly outside the GC. LMP1 by itself could recapitulate almost one-quarter of the transcriptional changes observed in HL cells [24]. Mice expressing LMP1 developed B cell lymphomas, but those expressing LMP2A had phenotypically normal BCR-positive B cells and did not show an increased propensity for lymphoma development [25].

The interactions between EBV and host microenvironment are relevant not only for the establishment of EBV latent infection in B cells but also for the development of EBV-associated lymphomas. Individuals latently infected by EBV have mounted adaptive, T cell-dependent anti-viral immune responses that keep the number of infected host cells low, in order to prevent the development of EBV-driven malignancies [26]. In EBV-associated HL, the presence of EBV elicits a higher number of infiltrating regulatory CD4+ T cells (Tregs), that secrete IL10, and activated cytotoxic CD8+ T cells [11]. In addition, significantly higher numbers of tumor-associated macrophages are observed in EBV+ HL as compared to EBV-negative HL [27], which can also produce IL10 [26]. In pediatric HL, EBV+ cases were characterized by a more intense T cell infiltrate, exhibiting a cytotoxic and Th1 profile and higher CD68+ cell counts [28,29]. Furthermore, an age-related influence of the immune system was suggested to modulate the tumor microenvironment in pediatric HL [28]. Given the fact that EBV-associated malignancies can occur if tumor precursor cells have escaped from immune response, comparative studies of the microenvironment composition between EBV pediatric asymptomatic carriers and EBV-associated pediatric lymphomas were performed. Therefore, CD4, CD8, Foxp3, GrB, IL10, and CD68 immune cell markers were studied in order to explore if differences around EBV-infected CG cells and EBV+ tumor cells derived from GC cells could be involved in the malignant transformation.

Even though a statistical difference in CD8+ and GrB+ cell responses were demonstrated in both EBV-associated pediatric HL and DLBCL [28,30], the recruitment of cytotoxic T cells was not observed in this series either in HL nor around EBV-infected GC cells when EBV+ and negative cases were compared. In tonsils from IM patients, a prevalence of CD8+ T cells was proved [31], whereas in established EBV infection, at least 20% of the CD8+ T cell population is specific for EBV in some tonsils, mostly against latent antigens [32], but there are preferentially located at the subepithelial region, not at the GC [15,33].Therefore, CD8+ T cell response may be mostly involved in the control of EBV infection in this specific region. Nevertheless, in EBV-associated HL both cytotoxicity markers, CD8 and GrB, were increased compared to EBV+ GC, but this difference was not observed when CD8 and GrB cell counts were compared in EBV− HL and GC, suggesting a collaborative influence of EBV in the HRS tumor cells to recruit cytotoxic immune cells. An increase in regulatory T cells was described in the tumor infiltrates of EBV− associated HL patients [34]. In contrast, the presence of this regulatory T cells was proved in IM and control tonsils, but accompanied by a decrease of this population in the blood of IM patients [35].Therefore, the role of regulatory cells in controlling primary EBV infection to a subclinical level was suggested, and IM may represent a failure of this protective mechanism [35]. In EBV+ HL, markers of suppression such as IL10 are also augmented, suggesting, on one hand, that the expression of viral proteins in these cells could enable their escape from the virus-specific cytotoxic T-cell response, and, on the other hand, that the signs of immune exhaustion in patients who develop lymphomas could be related to the increased soluble IL10 levels [34,36]. Even though no change in Foxp3+ cells was proved in between EBV+ and EBV− HL and tonsillar GC, an increase in IL10+ cell counts around EBV+ cells was observed in both HL and GC. The cytotoxic environment around EBV+ HRS cells could not be effective to control EBV driven malignancy, perhaps as a consequence of IL10 presence in EBV-associated HL, which turns into the coexistence of a cytotoxic with an immune regulatory milieu. Therefore, the increase of regulatory response in pediatric patients undergoing subclinical infection persists during the transformation process in order to regulate the cytotoxic immune response in EBV-associated HL.

Alternatively, CD4 and CD68 responses showed differences when tonsillar GC and GC derived HL in pediatric patients were compared. LMP1 and LMP2A synergistically drive aggressive GC B cell lymphoproliferative disease in a T− and NK cell–suppressed mouse model, suggesting that both viral antigens are necessary for the induction of fatal lymphoproliferative disease [37]. Furthermore, in a mouse model of acute EBV infection, when LMP1 was expressed, either alone or together with LMP2A at early GC B cells, those cells were under efficient T cell immunosurveillance. Depletion of T cells led to fatal expansion of LMP1/LMP2A co-expressing GC B cells, but this was not the case for LMP1 or LMP2A single-expressing GC cells [38]. A higher CD4+ T cell response was proved specifically at EBV+ tonsillar GC, which may indicate successful recruitment to control viral infection, and perhaps avoid B cell transformation, based on the fact that GC cells are the normal counterpart of the HRS cells, and most B cell non-Hodgkin lymphomas. The inefficient recruitment of CD4+ T cells in the context of EBV+ pediatric HL may represent a failure of this process to control EBV−mediated transformation. However, it seems like LMP1 is sufficient to trigger CD4+ T cell recruitment, given that, in one hand, LMP1 cell counts were correlated with CD4+ cell numbers at the GC, and, on the other hand, no differences in CD4+ T cell counts were observed when LMP1 was expressed with LMP2A and/or EBNA2 at the GC, as well as in LMP1+/LMP2A+ HL cases. Macrophage response in the context of EBV infection has been studied mostly in EBV-related malignancies. Concerning pediatric HL, higher CD68+ cell counts were described in EBV-associated pediatric HL, but without impact on survival [29]. In pediatric tonsils, an increase in CD68+ cell number was reported in EBV+ zones [15]. Despite the increase in CD68+ cells observed in the GC in pediatric tonsils of this series, this rise was not sustained in EBV-associated pediatric HL. Therefore, local cooperation between CD4 and CD68 to control EBV infection and perhaps transformation can be suggested at the GC. This cooperation may be reduced or lost under the transformation process triggered by EBV toward HL.

In summary, as far as we know, this is the first study comparing viral characteristics and microenvironment composition in tonsils from pediatric carriers and an EBV-associated GC-derived lymphoma. The genes downregulated in EBV+ HRS cells include those involved in TLR signaling and T cell co-stimulation, suggesting that evasion of innate and adaptive immunity could be more important in EBV-positive HL [25]. Our findings point us out to suggest that, even though EBV expression is restricted to a small number of cells at the GC compared with positive cells outside this histological structure, LMP1 expression may be sufficient to drive neoplastic transformation. In addition, the immune regulatory environment in pediatric patients may act to regulate the cytotoxic immune response in EBV-associated HL. Finally, CD4+ and CD68+ cells may be recruited to act in a local collaborative way to restrict, at least in part, viral-mediated lymphomagenesis. 

## 4. Materials and Methods

### 4.1. Patients and Samples

This study was conducted in two cohorts of patients: 60 children (2–16 years, median 9) diagnosed with HL at the Oncology Division, Ricardo Gutierrez Children’s Hospital; and 55 children (2–14 years, median 7) undergoing routine tonsillectomy previously diagnosed with tonsillar hyperplasia at the Otorhinolaryngology Service, Ricardo Gutierrez Children’s Hospital. Formalin-fixed paraffin-embedded (FFPE) samples were selected for the present study. Institutional guidelines regarding human experimentation were followed, according to the Helsinki Declaration of 1975. The protocol was approved by the Ethical Committee of Ricardo Gutierrez Children Hospital (code CEI 17.24), and written informed assent and consent was obtained from all patients or patient´s parents depending on age.

### 4.2. EBERs In Situ Hybridization

EBERs in situ hybridization (ISH) was performed on FFPE tissue sections using fluorescein isothiocyanate (FITC)-conjugated EBERs oligonucleotides as probes (Dako, Carpinteria, CA, USA). A monoclonal antibody anti-FITC labeled with alkaline phosphatase was used for the detection of hybridized sites (Dako), with NBT-BCIP as a substrate for the enzyme, according to manufacturer’s instructions. The cases were visualized at a visible spectrum.

### 4.3. LMP1, LMP2A, and EBNA2 Immunohistochemistry (IHC)

IHC for LMP1 (mAb CS1–4, Dako), LMP2A (clone 15F9, Abcam, Cambridge, UK), and EBNA2 (clones 1E6 and R3, kindly provided by Dr. Kremmer, Institut fur Immulogie, Munchen, Germany) were used to detect and localize EBV latent protein expression in tonsils and HL biopsies. Antigen unmasking with sodium citrate buffer (pH 6) in a microwave oven for 10 min was performed. All antibodies were incubated overnight at 4 °C. IHC detection primary antibody was carried out using a universal streptavidin–biotin complex-peroxidase detection system (UltraTek HRP Anti-Polyvalent Lab Pack, ScyTek, West Logan, UT, USA) according to the manufacturer’s instructions. In the tonsillectomies, LMP1 expression was employed in order to discriminate EBV+ and EBV-GC in all cases, given the fact that we have previously observed LMP1+ expression in all EBERs+ cases [10]. Once determined, LMP2A and EBNA2 expression and microenvironment compositions around infected and noninfected GC were compared.

### 4.4. Immune Cell Detection and Cell Counting

IHC was performed on 4µm serial FFPE tonsil and HL biopsy sections to characterize cell populations with the following antibodies: CD8 for cytotoxic T lymphocyte (CTL) (clone SP57, Ventana Roche), Granzyme B (GrB) for activated cytotoxic cells (clone GB7, AbDSerotec, Oxford, UK), CD68 for macrophages (clone KP-1, Ventana Roche), Foxp3 for regulatory T lymphocytes (Treg) (Abcam, Cambridge, UK), IL10 for anti-inflammatory cytokine productive cells (Abcam), and CD4 for T helper (Th) (Ventana Roche).

All cell markers were observed and counted by two pathologists in serial slides on the basis of the best-preserved areas around EBV+ and EBV− zones in both HL and tonsil samples. The results were expressed as immunopositive cells number/total cell number.

### 4.5. Statistical Analysis

Statistical analysis was performed using GraphPad Prism 4 software (GraphPad Software Inc., San Diego, CA, USA). Categorical variables were analyzed using Fisher’s exact test. Mann–Whitney and Wilcoxon matched-pairs tests were used to compare the means between T cell population markers in relation to EBV presence. Correlations between data were determined by employing Pearson’s rank correlation index. All tests were two-tailed, and a *p* < 0.05 was considered statistically significant.
